# Use of placental vascularization indices and uterine artery peak systolic velocity in early detection of pregnancies complicated by gestational diabetes, chronic or gestational hypertension, and preeclampsia at risk

**DOI:** 10.3325/cmj.2017.58.161

**Published:** 2017-04

**Authors:** Ábel T Altorjay, Andrea Surányi, Tibor Nyári, Gábor Németh

**Affiliations:** 1 Department of Obstetrics and Gynecology, University of Szeged, Faculty of Medicine, Szeged, Hungary; 2 Department of Medical Physics and Informatics, University of Szeged, Faculty of Medicine, Szeged, Hungary

## Abstract

**Aim:**

We aimed to investigate correlations between uterine artery peak systolic velocity (AUtPSV), and placental vascularization in groups of normal blood pressure (NBP) and hypertensive disorders of pregnancy (chronic hypertension (CHT), gestational hypertension (GHT) and preeclampsia (PE)) alone or in combination with gestational diabetes mellitus (GDM), and hypothesized that AUtPSV rises when GDM complicates pregnancy hypertension.

**Methods:**

Placental 3-dimensional power Doppler indices, such as vascularization index (VI), flow index (FI), and vascularization-flow index (VFI), and uterine artery peak systolic velocity (AUtPSV) were measured in CHT (N = 43), CHT+GDM (N = 15), GHT (N = 57), GHT+GDM (N = 23) and PE (N = 17) pregnancies, and compared to NBP (N = 109). Correlations were analyzed between vascularization indices, AUtPSV, pregestational BMI and adverse pregnancy outcome rates.

**Results:**

In our results VI was higher in CHT (*P* = 0.010), while FI was lower in CHT (*P* = 0.009), GHT and PE (*P* = 0.001) compared to NBP. In case of VFI, significant difference was found between CHT and GHT (*P* = 0.002), and NBP and PE (*P* = 0.001). FI was found prognostic for umbilical pH and neonatal birth weight. Pre-gestational BMI was significantly higher in GHT+GDM compared to GHT, and in CHT+GDM compared to the CHT group. As for AUtPSV, significant difference was found between NBP and CHT (*P* = 0.012), NBP and CHT+GDM (*P* = 0.045), NBP and GHT+GDM (*P* = 0.007), NBP and PE (*P* = 0.032), and GHT and GHT+GDM (*P* = 0.048) groups.

**Conclusion:**

Our study revealed that vascularization indices and AUtPSV show significant differences due to gestational pathology, and can be useful in detection of pregnancies at risk.

Hypertension during pregnancy occurs in 5 - 7% of all pregnancies, and about 70% of them occur in first-time pregnancies (1). Chronic hypertension (CHT) is a complication in 1%-2% of all pregnancies, while gestational hypertension (GHT) is a complication in 3%-6% of all pregnancies (1-3). It can progress to preeclampsia (PE) in about 25% of cases, especially when hypertension presents before 32 weeks of gestation (4). In Hungary, the prevalence of chronic hypertension among pregnant women is 1%-2%, that of gestational hypertension is 3%-6% (3), and the prevalence of gestational diabetes mellitus (GDM) is 6%-9.5% (5).

Pregnancy hypertension is a leading cause of maternal and fetal morbidity and mortality; furthermore its pathophysiological background is poorly understood (6).

Proper uterine and placental vascularization is important for the normal development of pregnancies (7,8). Pathological fetomaternal circulation accompanying pregnancy hypertension can lead to elevated resistance in uterine circulation, which can cause placental insufficiency and decreased fetal oxygenation (9), as a result of the pathological changes in placenta. In diabetic pregnancies, the placenta also has morphological changes that may result in reduced uteroplacental perfusion (10,11). Placental hystological maldevelopment can result in premature birth, intrauterine hypoxia, or even intrauterine death (12). These morphological changes can be detected indirectly by ultrasound in the form of vascularization indices (13).

Improvements in three-dimensional (3D) ultrasound have allowed us to obtain and study volumes from different organs more precisely (14), and color map provided by power Doppler has made the study of vessels with low resistance much easier (15). Combining these techniques has enabled us to study the morphology of the vascular tree and to quantify the direct blood flow of the placenta (13,16).

Differences between placental vascularization indices in case of pregnancy hypertension complicated with GDM were unknown. It was also uclear whether there is difference in AUtPSV values depending on the presence of GDM, or not in case of pregnancy hypertension.

We hypothesized that placental vascularization and AUtPSV will differ depending on pregnancy pathology (CHT; GHT; GDM combined with CHT or GHT; and PE).

The purpose of the present study was to clarify the effect of using uterine artery peak systolic velocity (AUtPSV) measurements, 3D ultrasound, and placental vascularization indices on hypertensive disorders of pregnancy and hypertensive disorders of pregnancy complicated with GDM in second and third trimesters.

## PATIENTS AND METHODS

We performed a prospective Doppler study: placental vascularization indices and uterine artery peak systolic velocity. We included women with singleton pregnancies seen once in the second or third trimester at our outpatient clinic at University of Szeged, Faculty of Medicine, Department of Obstetrics and Gynecology, in Szeged. Our study was carried out between 1 March 2014 and 31 March 2015 in accordance with the Code of Ethics of the Declaration of Helsinki for scientific research involving humans, and our study was approved by the institutional research ethics committee (No.: 32/2014). Informed consent was signed by the observed person after a detailed and clear explanation about the conditions and aims of the survey.

### Patients

#### Inclusion criteria

In our study we analyzed singleton pregnancies between 20-38 weeks of gestation, which were divided into six groups as you can see in Table 1. Gestational age was determined on the basis of the first day of the last menstrual period and on the basis of the first trimester ultrasound biometry (biparietal diameter (BPD) and crown-lump length (CRL)).

**Table 1:** Groups of pregnant women and the number of cases examined

Groups of pregnant women		No.of cases
NBP	group of pregnant women with normal blood pressure	109
CHT	group of pregnant women with chronic hypertension	43
CHT+GDM	group of pregnant women with chronic hypertension complicated with gestational diabetes mellitus	15
GHT	group of pregnant women with gestational hypertension	57
GHT+GDM	group of pregnant women with gestational hypertension complicated with gestational diabetes mellitus	23
PE	group of pregnant women with pre-eclampsia	17

#### Diagnostic criteria for hypertension

High blood pressure (>140 mm Hg systolic or >90 mm Hg diastolic) was defined on the basis of the International Society for the Study of Hypertension in Pregnancy (ISSHP) (4).

Blood pressure was measured (BP A100 PLUS, Microlife AG, Windau, St. Gallen, Switzerland) three times on each occasion, and patients were scheduled for a check-up every two weeks.

Patients in the case groups had ongoing antihypertensive therapy with oral alpha-methyldopa 250 mg (Dopegyt, EGIS Pharmaceuticals 105 PLC., Budapest, Hungary) and they had dietary salt restrictions according to the Hungarian guidelines (17).

Inclusion criteria for CHT was high blood pressure pre-dating pregnancy. As many women did not have their blood pressure measured before the pregnancy, we relied on the first trimester blood pressure according to ISSHP when defining high blood pressure in these women. Inclusion criteria for GHT was new onset of hypertension after 20 weeks of gestation, for which it was important to have normal blood pressure documented either before the pregnancy or at least in early pregnancy before pregnancy-related decrease in blood pressure occurred (4).

#### Diagnostic criteria for preeclampsia

In the diagnosis of PE we applied the definition of ISSHP (4), which defines PE as a combination of new onset of hypertension, that occurs after 20 weeks of gestation and the coexistence of one or more of the following new-onset conditions such as proteinuria, other maternal organ dysfunctions (renal insufficiency, liver involvement, neurological or hematological complications) and uteroplacental dysfunction in the form of fetal growth restriction (IUGR).

#### Diagnostic criteria for GDM

GDM was defined on the basis of the recommendation of the World Health Organization (WHO) 2013 (18). In Hungary patients are screened with an oral glucose tolerance test (OGTT) containing 75 mg oral carbohydrate on the basis of WHO guidelines. Groups CHT+GDM and GHT+GDM consisted of patients who had dietary sugar and salt restriction in addition to the antihypertensive therapy. Their blood glucose and HgA1c levels were in normal range. Those patients, in which introduction of insulin therapy was needed, were excluded from the study. All pregnant women with GDM had normal glycemic response. We used HgA1c for GDM follow up. Body mass index (BMI) was defined as follows: underweight: <19 kg/m^2^; obese: >30 kg/m^2^.

### Methods

#### Ultrasound diagnosis

Pregnant women who visited our outpatient clinic had been prospectively enrolled into 6 groups as follows: a control group (N = 109) and 5 case groups (N = 155). Those who did not meet the criteria of the study design during the study period were excluded (N = 612).

An initial 2D conventional ultrasound study provided data about fetal position and presentation, body movements and fetal heart rate, placental localization, umbilical cord insertion, and volume of amniotic fluid. All patients were scanned in a semi-recumbent position.

Examination started with fetal biometry in 2D mode ultrasound to assess biparietal diameter (BPD), head circumference (HC), abdominal circumference (AC), femur length (FL) and to calculate estimated fetal weight (EFW) with the help of formula B of Hadlock (19), followed by color Doppler study of the uterine arteries (20).

**Figure 1:**
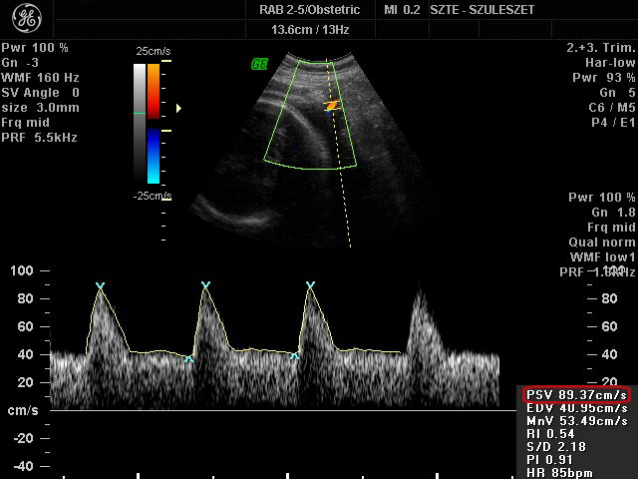
Measurement of uterine artery peak systolic velocity (AUtPSV) taken from the sagittal section of the uterus with color flow mapping and applied pulsed wave Doppler. PSV value is highlighted in red.

To identify the uterine arteries, we obtained a sagittal section of the uterus, and used color flow mapping for AUtPSV (cm/s) (Figure 1). Then we applied pulsed wave Doppler with the sampling gate set at 2 mm. The angle of insonation was <30^◦^ and we recorded at least three consecutive uniform waveforms (21). The impact of placentation on AUtPSV was ruled out by calculating the average value of the left and right uterine artery.

The next step was the 3D scan of the placenta at the insertion point of the umbilical cord. We used 3D rendering mode, in which the color and gray value information was processed and combined to give a 3D image (mode cent: smooth, 4/5; FRQ, low; quality, 16; density, 6; enhance, 16; balance, 150; filter, 2; actual power, 2 dB; pulse repetition frequency, 0.9) (22). Power Doppler window (pulse repetition frequency at 900 Hz and wall filter of 50 Hz) was placed over the placenta, mapping the vascular tree from basal to chorionic plates, as this technique shows higher sensitivity because it is based on amplitude instead of mean frequencies to depict the vascular tree (15). Moreover, color mapping is independent of the angle of insonation and does not show ‘aliasing’. However, it is more sensitive to patient movements, so the volumes should be acquired while avoiding any probe or patient movements; otherwise artifacts could be present.

The 3D static volume box was placed over the highest villous vascular density zone at umbilical cord insertion (23). The sweep angle was set at maximum 70°. The three planes of the acquired placental volume were explored to localize the zone where the highest vascular density was found by power Doppler mode. Volume acquisition was made during a time interval varying from 5 to 10 s in the absence of fetal movements and with the mother staying as still as possible. We used fast low resolution acquisition to avoid any kind of artifacts. The variation in acquisition time was also dependent on the size of the volume box, and it correlated with the age of gestation.

All 2D and 3D ultrasound scans were performed using Voluson 730 system (RAB 2-5 MHz transducer, GE Healthcare, Kretztechnik, Zipf, Austria). The same pre-established instrument power settings were used in all cases (‘Obstetrics/2–3 trimester’ in 2D mode).

#### Calculation of 3-dimensional power Doppler indices

Volume files were analyzed using the virtual organ computer-aided analysis (VOCAL) program pertaining to the computer software 4D VIEW (GE Medical Systems, Austria, version 10.4) by an expert in 3D analysis.

We used Mercé-type sono-biopsy (23), a reproducible, valid alternative for evaluation of the vascular tree of the entire placenta (24,25). The spherical sample volume was 28 mL constantly, and the VOCAL software automatically calculated the color scale values in a histogram, and the vascularization indices (vascularization index (VI), flow index (FI) and vascularization flow index (VFI)) from the acquired spherical sample volume in all cases (23).

The 2D and 3D ultrasound acquisitions were performed at the same time, and 3D volume files were analyzed by VOCAL at a later time. The ultrasound images of 2D and 3D scans were stored on a hard disk (HD).

#### Data collection after delivery

Data about neonatal outcome and mode of delivery (normal delivery, planned cesarean section or acute cesarean section) were collected and classified after delivery. We collected data on 1-, 5-, and 10-minute Apgar scores, umbilical pH, neonatal birth weight, rate of neonatal complications such as apnea, polycythemia, hypoglycemia, respiratory distress syndrome, dysmaturity and feeding difficulties, and the rate of macrosomia and premature birth.

### Statistical analysis

Statistical analyses were performed with IBM SPSS Statistics 21.0 for Windows program (IBM, New York, USA). Kolmogorov-Smirnov test results were significant for our database demonstrating that our study samples were not normally distributed. Continuous variables were expressed as median ± standard deviation. The Kruskal-Wallis test was used for the comparison of continuous variables in the six groups examined, whereas comparison between the pathological groups was performed with Mann-Whitney U test in case of vascularization indices, and with Bartlett’s test with Bonferroni’s modification in case of AUtPSV, (level of significance was set at *P* < *.05*). Univariate comparisons for categorical variables were assessed with χ^2^ tests. Linear regression coefficient values and equations depending on gestational age were also calculated for VI, FI, VFI and AUtPSV for all pathological and control groups. The association between placental 3D power Doppler indices, AUtPSV, pre-gestational BMI, neonatal birth weight and pregnancy outcome, 2-D color Doppler indices (PIs of umbilical and uterine arteries) was determined by Spearman's rank correlations.

## RESULTS

The analysis of 3D volume acquisition demonstrated that VI indices are significantly higher in CHT (*P* = 0.010), CHT+GDM (*P* = 0.054), and GHT+GDM (*P* = 0.973) groups compared to NBP (Table 2). In case of GHT, VI was lower (*P* = 0.152) compared to NBP, though the difference was not significant. There was significant difference between the CHT and GHT groups (*P* = 0.010). All PE cases evolved from GHT cases, therefore we analyzed the changes in 3-DPD indices, but the difference between GHT and PE groups in case of VI was not statistically significant (*P* = 0.175).

**Table 2:** Three-dimensional Power Doppler indices: vascularization index (VI), flow index (FI), and vascularization flow (VFI) index, and uterine artery peak systolic velocity (AUtPSV) in pregnancies with normal blood pressure (NBP), chronic hypertension (CHT), chronic hypertension and gestational diabetes mellitus (CHT+GDM), gestational hypertension (GHT), gestational hypertension and gestational diabetes mellitus (GHT+GDM), and preeclampsia (PE)

	Groups of pregnant women					
Parameter	CHT+GDM (n = 15)	CHT (n = 43)	NBP (n = 109)	GHT (n = 57)	GHT+GDM (n = 23)	PE (n = 17)
**VI** (%, mean±SD)	5.3 ± 3.5	14.4 ± 10.1	10.4 ± 6.2	7.7 ± 7.1	5.4 ± 2.4	4.9 ± 3. 2
**FI** (mean±SD)	42.8 ± 9.6	41.5 ± 8.2	46.1 ± 7.6	38.5 ± 9.6	36.4 ± 8.3	36.5 ± 5.7
**VFI** (mean±SD)	2.3 ± 1.5	3.6 ± 2.8	4.1 ± 2.5	3.0 ± 2.5	2.0 ± 1.1	2.0 ± 1.6
**AUtPSV** (cm/s, mean±SD)	45.3 ± 14.1	50.0 ± 16.6	59.5 ± 23.1	56.8 ± 18.4	68.6 ± 30.9	52.4 ± 13.0

In comparison with NBP group, FI was significantly lower in CHT (*P* = 0.009), CHT+GDM (*P* = 0.010), GHT (*P* < 0.001), GHT+GDM (*P* < 0.001), and PE (*P* < 0.001) groups; however, there was no statistically significant difference between CHT and CHT+GDM (*P* = 0.354); GHT and GHT+GDM (*P* = 0.443); and GHT and PE (*P* = 0.183) groups.

For VFI, no significant difference was found between CHT and CHT+GDM (*P* = 0.073) or NBP (*P* = 0.973), GHT and GHT+GDM (*P* = 0.428), GHT and PE (*P* = 0.128) or GHT and NBP (*P* = 0.075). Statistically significant difference can only be described when PE was compared to NBP (*P* = 0.010).

AUtPSV showed significant difference between NBP and CHT (*P* = 0.012) and GHT and GHT+GDM (*P* = 0.048) groups, but not between CHT and CHT+GDM (*P* = 0.062), GHT and NBP (*P* = 0.087), and GHT and GHT+GDM (*P* = 0.320).

Pre-gestational BMI was significantly higher (*P* = 0.009) in GHT+GDM (33.46 ± 7.11) compared to GHT (30.37 ± 5.80), as well as in CHT+GDM (32.84 ± 3.64) compared to the CHT (30.55 ± 5.68) group. Strong positive linear correlation was found between VI (*P* = 0.009), FI (*P* = 0.007) and neonatal birth weight (BW). Mean FI was 45.7 in case of normal pregestational BMI and 41.2 in case of elevated BMI, thus elevated pregestational BMI had substantial influence on FI depression (*P* = 0.048) and fetal growth development, consequently on BW.

**Figure 2:**
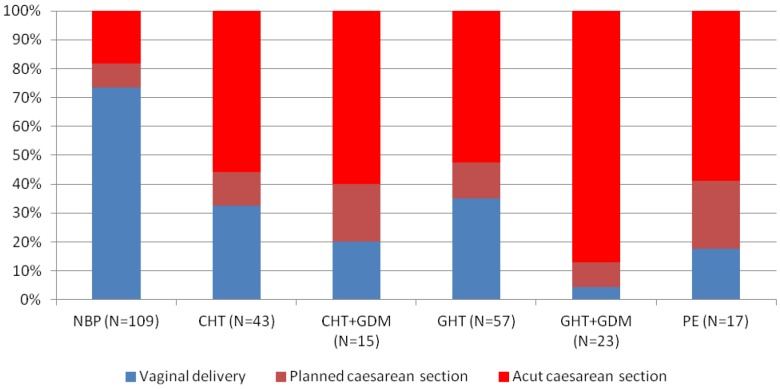
Mode of delivery in pregnancies with normal blood pressure (NBP), chronic hypertension (CHT), chronic hypertension and gestational diabetes mellitus (CHT+GDM), gestational hypertension (GHT), gestational hypertension and gestational diabetes mellitus (GHT+GDM) and preeclampsia (PE).

**Figure 3:**
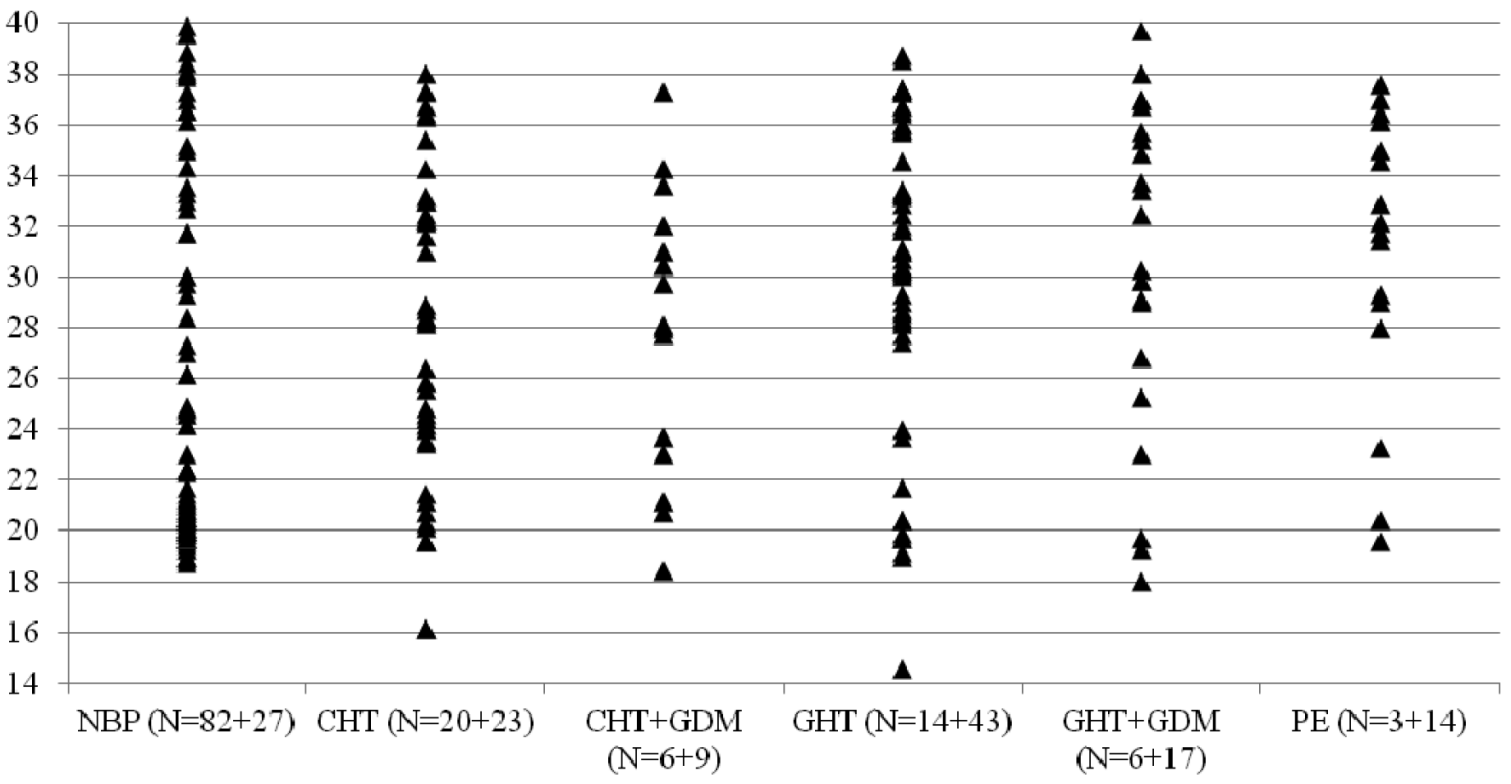
Weeks of gestation at the time of 3D scan in pregnancies with normal blood pressure (NBP), chronic hypertension (CHT), chronic hypertension and gestational diabetes mellitus (CHT+GDM), gestational hypertension (GHT), gestational hypertension and gestational diabetes mellitus (GHT+GDM) and preeclampsia (PE). (N = N2+N3; N2: second trimester, N3: third trimester).

Maternal and perinatal characteristics and pregnancy outcomes included the maternal age, physique, lenght of gestation, the neonatal biophysical parameters, adaptation parameters and complication of intrauterine chronic hypoxia (Table 3). We also analyzed mode of labor (Figure 2) and gestational ages at 3-DPD ultrasound investigations (Figure 3) with respect to blood pressure and other investigated patology.

**Table 3:** Maternal and fetal characteristics in pregnancies with normal blood pressure (NBP), chronic hypertension (CHT), chronic hypertension and gestational diabetes mellitus (CHT+GDM), gestational hypertension (GHT), gestational hypertension and gestational diabetes mellitus (GHT+GDM) and preeclampsia (PE)

		Groups of pregnant women					
Parameter		CHT+GDM (n = 15)	CHT (n = 43)	NBP (n = 109)	GHT (n = 57)	GHT+GDM (n = 23)	PE*(n = 17)
Mean maternal age (years; mean±SD)		34.4 ± 1.4	32.8 ± 4.2	30.7 ± 4.7	31.1 ± 5.4	32.5 ± 6.3	29.5 ± 5.2
*P*		0.023*				0.037*	
Weeks of gestation at the time of 3D scan (mean±SD)		28^+1^±7^+2^	28^+5^±6^+6^	24^+6^±7^+2^	31^+6^±6^+4^	29^+6^±7^+5^	31^+2^±7^+1^
Weeks of gestation at the time of delivery (mean±SD)		37^+4^±1^+5^	38^+5^±1^+1^	38^+3^±1^+6^	38^+0^±2^+2^	37^+1^±2^+3^	36^+6^±3^+5^
Pregestational BMI (kg/m^2^; mean±SD)		32.8 ± 3.6	30.8 ± 4.6	30.7 ± 5.2	31.0 ± 5. 0	33.4 ± 7.1	27.2 ± 6.10
*P*		0.041*				0.047*	
Number of women with pregestational maternal obesity (BMI over 30)		9/15	25/43	18/109	14/57	10/23	7/17
Weight gain during pregnancy until delivery (kg; mean±SD)		7.75 ± 7.02	8.45 ± 4.52	7.86 ± 4.95	13.44 ± 5.77	14.50 ± 4.53	11.41 ± 3.49
*P*					<0.001*	<0.001*	0.011*
Number of premature births		1/15	3/43	8/109	7/57	5/23	8/17
Apgar score (mean±SD)	1-minute	8.30 ± 0.75	8.93 ± 1.09	8.86 ± 0.57	8.69 ± 1.52	8.21 ± 1.75	8.14 ± 1.43
	5-minute	9.53 ± 0.66	9.68 ± 0.84	9.50 ± 0.67	9.65 ± 0.68	9.39 ± 1.15	9.29 ± 1.13
	10-minute	9.93 ± 0.27	9.91 ± 0.35	9.91 ± 0.21	9.82 ± 0.60	9.65 ± 1.07	9.55 ± 0.84
Umbilical pH (mean±SD)		7.29 ± 0.05	7.23 ± 0.07	7.27 ± 0.08	7.26 ± 0.07	7.25 ± 0.07	7.25 ± 0.07
Number of perinatal compli-cations	Apnea	7/15	3/43	9/109	6/57	5/23	7/17
	*P*	<0.001*			0.001*		<0.001*
	Polycythemia	0/15	2/43	7/109	7/57	0/23	2/17
	Hypogly-cemia	6/15	1/43	2/109	2/57	4/23	3/16
	*P*	0.012‡			0.022§		
	Respiratory distress syndrome	0/15	0/43	0/109	0/57	2/23	3/16
	Dysmaturity^II^	0/15	0/43	3/109	4/57	1/23	12/17
	Feeding difficulties	6/15	1/43	7/109	4/57	5/23	0/17
	*P*	<0.001*				0.002*	
Neonatal birth weight (grams; mean±SD)		3130 ± 296	3377 ± 374	3346 ± 555	3236 ± 751	3601 ± 811	2422 ± 817
Rate of macrosomia (neonatal birth weight over 4000g)		2/15	2/43	12/109	3/57	1/23	0/17

*All cases were intrauterine growth restriction.

^†^vs normal blood pressure (NBP) group.

‡CHT+GDM vs CHT.

§GHT vs GHT+GDM.

^II^Syndrome of an infant born with relative absence of subcutaneous fat, wrinkling of the skin, prominent finger and toe nails, and meconium staining of skin and placental membranes; often associated with postmaturity or placental insufficiency.

Interestingly the highest cesarean section rate was observed in the GHT+GDM group, even in the PE group the rate of normal delivery was three times higher than in GHT+GDM, although premature birth rate was the highest in PE. All PE cases were consisted of patients with GHT and IUGR. As for adverse pregnancy outcome rates, FI was found prognostic for umbilical pH and neonatal birth weight.

In the assessment of neonatal outcomes rate of apnea it was significantly elevated in GHT patients (*P* = 0.042) compared to CHT patients and it was more significantly elevated in GHT+GDM (*P* < 0.001), CHT+GDM (*P* < 0.001) and PE (*P* = 0.001) cases. One other characteristic perinatal complication specific to newborns of diabetic mothers, hypoglycemia was also significantly higher in CHT+GDM (*P* = 0.012) compared to CHT, and GHT+GDM (*P* = 0.022) compared to GHT.

## DISCUSSION

In our in-vivo study of vascularization analysis we described examined the effect of GDM on placental vascularization and AUtPSV in pregnancies complicated with pregnancy hypertension. We found no correlation between placental vascularization indices (VI, FI and VFI) or AUtPSV findings and maternal age or weeks of gestation.

Because of the chronic intrauterine hypoxia the placental alterations are presented from first trimester Similarly to previous first trimester findings (26). In case of hypertensive disorders of pregnancy (CHT, GHT or PE) placental vascularization, which is characterized by FI, is significantly lower compared to NBP cases in the second and third trimesters, and it becomes even more affected when hypertension is associated with gestational diabetes (CHT+GDM, GHT+GDM) and correlated with the lenght of pathological lenght of gestation. On the other hand, according to AUtPSV findings, GDM aggravates CHT and GHT hypoxic damage of placentas and fetuses. AUtPSV is an absolute speed value indicates vascular missing adaptation more sensitively than traditional flow indices, such as Systolic-Diastolic ratio (S/D) or Pulsatility Index (PI).

The increased AUtPSV of diabetic women demonstrated in our study reflects changes in systemic arteriolar placental afterload, myocardial contractility, heart rate and preload. The placenta in diabetic pregnancy has morphological changes that may result in reduced uteroplacental perfusion. The absence of a difference in uterine artery PI values between fetuses of diabetic women and normal controls argues against a modification increased arterial compliance may increase AUtPSV without altering afterload (27).

Most studies represent normal Doppler flow in the uteroplacental circulation in pregnancies complicated by maternal DM, except in those cases complicated by PE (28).

Where women had pre-existing hypertension (CHT cases) and normally functioning placenta, we detected no significant difference in adverse pregnancy outcome rates between CHT and NBP groups despite the lower FI rate, though VI was found to be significantly higher in CHT. In GHT and PE group, both VI and FI were significantly lower (26), as a proof of placental maldevelopment.

Only FI was found to be significantly lower in CHT+GDM compared to CHT, though we expected significantly lower results in case of VI, VFI as well based on an earlier study (22).

AUtPSV in CHT was significantly lower compared to NBP, which may be the result of the higher VI rate, and it may also confirm the existence of a placental response on elevated maternal blood pressure predating pregnancy. Interestingly, the GDM did not trigger the chronic hypoxic alteration in CHT group.

There was no significant difference in AUtPSV between GHT, PE and NBP, but AUtPSV was significantly higher in CHT+GDM compared to both CHT and GHT, which correlates with the higher adverse pregnancy outcome rates specific to gestational diabetes mellitus.

The highest mean maternal age was found in case of pregnancy hypertension complicated with GDM, highlighting the fact that higher maternal age is an important risk factor in GDM. Above it the GDM has effect on neonatal outcome, as well. GDM gives a higher rate for hypoglycemic episodes and feeding difficulties.

In case of PE there was no further increase in the rate of hypoglycemic episodes.

Macrosomia is most likely to occur in case of gestational diabetes. In case of NBP, it is possible that there were undiagnosed gestational diabetic patients in our NBP group. The lower macrosomia rate on the other hand in case of GHT+GDM compared to NBP or CHT+GDM might be the result of impaired placentation in case of GHT that prevented GHT+GDM cases end up with macrosomia in neonates.

There are several limitations of our study. The method is very sensitive and, therefore, we had to reduce the interobserver bias. We should apply standardized settings of the ultrasound equipment. Fetal movements and maternal respiratory movements result in artifacts in the 3-DPD records. Data recording and analyzing are time consuming, thus these kind of investigations are difficult to introduce into routine maternal care. The GDM screening is performed in first and second trimester based on WHO guideline, it is possible that there were undiagnosed gestational diabetic patients, which developed in third trimester.

Our study revealed that certain placental vascularization indices and AUtPSV may indicate on significant differences due to gestational pathology, thus placental vascularization indices and AUtPSV can be useful in the early detection of pregnancies at risk in order to possibly prevent complications, but extensive researches are requires to determine the clinical significance.
